# Social networks and infectious diseases prevention behavior: A cross-sectional study in people aged 40 years and older

**DOI:** 10.1371/journal.pone.0251862

**Published:** 2021-05-19

**Authors:** Lisanne C. J. Steijvers, Stephanie Brinkhues, Christian J. P. A. Hoebe, Theo G. van Tilburg, Vivian Claessen, Noortje Bouwmeester-Vincken, Femke Hamers, Petra Vranken, Nicole H. T. M. Dukers-Muijrers

**Affiliations:** 1 Department of Sexual Health, Infectious Diseases, and Environmental Health, Public Health Service South Limburg, Heerlen, The Netherlands; 2 Department of Knowledge and Innovation, Public Health Service South Limburg, Heerlen, The Netherlands; 3 Department of Medical Microbiology, Care and Public Health Research Institute (CAPHRI), Maastricht University Medical Center (MUMC+), Maastricht, The Netherlands; 4 Department of Sociology, Vrije Universiteit Amsterdam, Amsterdam, The Netherlands; 5 Department of Infectious Disease Control, Public Health Service Limburg North, Venlo, The Netherlands; 6 Department of Health Promotion, Care and Public Health Research Institute (CAPHRI), Maastricht University, Maastricht, The Netherlands; Universita degli Studi della Campania Luigi Vanvitelli, ITALY

## Abstract

**Background:**

Social networks, i.e., our in-person and online social relations, are key to lifestyle behavior and health, via mechanisms of influence and support from our relations. We assessed associations between various social network aspects and practicing behavior to prevent respiratory infectious diseases.

**Methods:**

We analyzed baseline-data (2019) from the SaNAE-cohort on social networks and health, collected by an online questionnaire in Dutch community-dwelling people aged 40–99 years. Outcome was the number of preventive behaviors in past two months [range 0–4]. Associations between network aspects were tested using ordinal regression analyses, adjusting for confounders.

**Results:**

Of 5,128 participants (mean age 63; 54% male), 94% regularly washed hands with water and soap, 55% used only paper (not cloth) handkerchiefs/tissues; 19% touched their face as little as possible; 39% kept distance from people with respiratory infectious disease symptoms; median score of behaviors was 2. Mean network size was 11 (46% family; 27% friends); six network members were contacted exclusively in-person and two exclusively via phone/internet. Participants received informational, emotional, and practical support from four, six, and two network members, respectively. Independently associated with more preventive behaviors were: ‘strong relationships’, i.e., large share of friends and aspects related to so called ‘weak relationships’, a larger share of distant living network members, higher number of members with whom there was exclusively phone/internet contact, and more network members providing informational support. Club membership and a larger share of same-aged network members were inversely associated.

**Conclusion:**

Friends (‘strong’ relationships) may play an important role in the adoption of infection-preventive behaviors. So may ‘weak relationships’, e.g. geographically more distant network members, who may provide informational support as via non-physical modes of contact. Further steps are to explore employment of these types of relationships when designing infectious diseases control programs aiming to promote infection-preventive behavior in middle aged-and older individuals.

## Background

The COVID-19 pandemic magnified mainstream recognition of the value of infection prevention strategies [1]. Behaviors, such as handwashing, cough hygiene, and keeping physical distance from people with respiratory infectious diseases symptoms, can prevent spread of pathogenic micro-organisms [2]. These are basic non-pharmaceutical preventive behaviors that people in the community can employ themselves, and thus are widely promoted, during the pandemic and likely also thereafter [2]. Therefore, effective preventive strategies to promote infection preventive behavior are very welcome.

Social networks have the potential to increase the effectiveness and reach of preventive strategies to promote various health behaviors [3, 4]. Social network aspects have been associated with smoking cessation, weight loss, and physical activity [3, 5–8]. Regarding pharmaceutical infection prevention behaviors, such as influenza and pneumococcal vaccinations, specific social network aspects as receiving informational support from family and friends [9], and older people who not live alone were found to be associated [10]. The role of social networks in non-pharmaceutical infection preventive behaviors has by our knowledge not yet been reported.

Social networks are all the offline and online social relationships (or: ties) that people have. Social network theory learns us that individuals tend to connect with others who are similar in terms of sociodemographic characteristics, age and sex, but also regarding behaviors and preferences, i.e., called homophily [11]. According to the theory of normative social behavior, network members are likely to share social norms and behavior [12]. Behavior, regardless of whether positive or negative, is modeled by and imitated from these similar others [13]. Further, social network members may support the ability to adopt healthy behaviors, by generating support to access health benefits [14] or by enacting on behavioral control [15].

The composition of social networks can be described by its structural aspects, such as the size and the diversity in roles of network members [16]. A person’s social network may contain many or only few relationships, who are close by or far away, or who are contacted in-person (i.e., physically face-to-face) or online/by telephone. Social networks include various types of relationships, such as family, friends, colleagues, or neighbors. Relationships might be further typed as strong or weak. Strong relationships are emotionally close, geographically close, or recurring interactions, such as family members, close-friends, or neighbors. Strong relationships may provide emotional support and practical support which can be important to make and maintain a healthy behavior change [17, 18]. Strong relationships are able to ‘control’ health behavior directly by facilitating resources or indirectly by introduction of norms and acting as ‘role models’ [15, 19, 20].

The strength of ‘weak relationships’ has been described by Granovetter [18]. Weak relationships are geographically, practically, or emotionally more distant, or typed by less recurrent interactions. Weak relationships can be very important as they play a crucial role in providing new ideas, influences and information e.g. knowledge, where to, how to, in providing informational support [21]. Weak relationships interact with strong relationships, and thereby expand a person’s social support network.

The ‘Social Network Assessment in Adults and Elderly’ (SaNAE) study aims to investigate social networks in relation to health and behavior, among middle aged and older adults in the Netherlands. As social relations can promote various healthy lifestyle behaviors, we hypothesize that social networks are able to promote infection prevention behaviors. Until now, data are lacking on which social network aspects play a role in the practice of non-pharmaceutical infection-preventive behaviors. Here, we examine this role of a wide-range social network aspects in the period just preceding the COVID-19 pandemic. In line with findings in vaccination behavior [9, 10], we hypothesize that informational support may also enable non-pharmaceutical infection preventive behavior, as well as the presence of positive role models, which can be family or friends.

We study behavior-outcomes that are effective in preventing respiratory infections, and that people can act on themselves, without requiring an interacting care provider (thus as opposed to pharmaceutical infection-prevention behaviors, as taking antibiotic prophylaxis or vaccination uptake). Findings may inform the strengthening of infection prevention strategies to employ the potential beneficial aspects of peoples’ social networks.

## Methods

### Ethics statement

This study was approved by the Medical Ethical Committee of the University of Maastricht (METC 2018–0698 and 2019–1035). Invitees were previous participants in the ‘Dutch Health Monitor’, which is a population-based survey of the Public Health Services South and North Limburg. Invitations were only sent to participants who had given permission (and their email-address) in the Dutch health monitor to be invited for future research. Before starting the questionnaire of the current SaNAE study, participants first gave electronic informed consent.

### Study population

The SaNAE study included participants between 40 and 99 years of age and living independently in the Dutch province of Limburg. In February-March 2019, we invited 11,728 persons by email, with a link to the online questionnaire. In total, 44% (n = 5,144) responded; non-respondents were on average four months older (p<0.001) and less often had a high educational level (32% versus 42%; p<0.001) than respondents, while the sex distribution was similar (p = 0.172) in respondents and non-respondents (S1 Table).

### Design

A cross-sectional study, with baseline data as part of the prospective SaNAE-cohort (www.sanae-study.nl).

### Measurements

We collected data on sociodemographic characteristics, chronic conditions, infection-preventive behaviors (here: outcome) and social network aspects (here: main determinants); see for an overview Table 1.

**Table 1:** Characteristics of the SaNAE study population (n = 5,128).

Individual characteristics	% (n) or mean (standard deviation, sd)
**Age (40–99); mean**	63.3 (10.3)
**Sex**	
Man	54.4 (2789)
Woman	45.6 (2339)
**Educational level**^**a**^	
Lower	23.1 (1187)
Medium	35.4 (1813)
Higher	41.5 (2128)
**Marital status**	
Married	76.6 (3927)
Single	15.6 (798)
Widowed	7.9 (403)
**Children**	
No	36.7 (1881)
Yes, children living at home	20.3 (1041)
Yes, children living not at home	43.0 (2206)
**Body Mass Index (BMI)**	
Underweight	0.7 (35)
Normal weight (BMI<25)	37.7 (1935)
Overweight (25< BMI >30)	42.1 (2158)
Obesity (≥30)	19.5 (1000)
**Mobility**	
No problems with daily activities	67.6 (3466)
Some problems with daily activities	30.4 (1561)
Not able to do daily activities	2.0 (101)
**Current smoking (yes)**	11.7 (598)
**Happiness score**	
Median [IQR: 7.0–8.0]	8.0 [7.0–8.0]
**Depression (PHQ-9 score ≥ 10)**	5.7 (290)
**Type II Diabetes Mellitus**	8.8 (450)
**Cardiovascular diseases**	17.3 (885)
**Asthma/COPD**	11.0 (565)
**Number of chronic conditions** ^**b**^	
None	66.6 (3414)
One	21.5 (1104)
Two or more	11.9 (610)
**Infection prevention behavior for respiratory infections**	
Wash hands regularly with water and soap	93.7 (4803)
Use only paper (not cloth) handkerchiefs/tissues	55.0 (2818)
Touch face as little as possible	18.8 (966)
Keep distance from people who have symptoms of respiratory infectious diseases	38.7 (1987)
**Infection prevention behavior for respiratory infections** ^**c**^	
Zero	3.4 (175)
One	26.8 (1376)
Two	40.7 (2088)
Three	18.2 (934)
Four	10.8 (555)
Mean sum score preventive measures for respiratory infections (sd)	2.1 (1.0)

^a^ Educational level is categorized into lower, medium and higher educational level. Lower educational level includes no education, primary education not completed, primary education and lower vocational education. Medium education level includes intermediate vocational education, higher secondary education. Higher educational level includes higher professional education and university education.

^b^ Include depression, type II DM, cardiovascular diseases, asthma/COPD

^c^ Preventive measures for respiratory infections include wash hands regularly with water and soap, use only paper tissues, touch face as little as possible and keep distance from people who have respiratory infection symptoms.

The respiratory-infection preventive behaviors assessed (yes/no) were current practice of ‘washing hands regularly with water and soap’, ‘using only paper (not cloth) handkerchiefs/tissues’, ‘touching one’s own eyes and nose (face) as little as possible’, and ‘keeping distance from people who have respiratory infectious diseases symptoms’. For statistical analyses, a variable was created to reflect the count of behaviors practiced (range zero to four).

Social networks were assessed by using a name generator, meaning that participants were asked to fill in the names of people who are important to them or provide support. Family, friends, acquaintances and other members were asked separate from each other and participants were able to fill in, up to fifteen family members, ten friends, ten acquaintances and five other members [22–24]. For each identified person, additional information such as sex or age was asked. For statistical analyses, the information was used to compute various social network aspects [24], reflecting structural and functional aspects, and that were proposed to reflect strong or weak relationships (described below).

#### Structural social network aspects

Network size was the total number of network members identified by a respondent. Four indicators for relationship type were the proportions, ranging from 0 to 100 percent, of network members who were family, friends, acquaintances, or other members. Proximity indicators were the proportions of network members who were household members, members who lived at walking distance, and more distant social relations, as people who lived less than half hour away by car, more than half hour away by car, or further away. A respondent without household members was defined as living alone. Homophily by sex indicated the proportion of network members with the same sex as the respondent. Homophily by age was the proportion of network members with the same age (within a five-year range). Contact with children was defined for children younger than five years. Mode of contact was assessed in the past two weeks, indicated by the number of network members who were contacted only in-person, only via telephone/internet, or via both modes. The number of network members was then categorized into four categories: zero members, one to two members, three to five members and six or more members. Network density was indicated by whether the respondents’ friends knew the respondents’ family. Lastly, social participation was assessed as club membership, i.e., sports clubs, religious or volunteer organizations, or talking, self-help, or internet groups.

#### Functional social network characteristics

Emotional social support was defined as the number of network members who provide the opportunity to discuss important matters. Informational social support was the number of network members who gave advice on problems. Practical social support was the number of network members who helped with small or larger jobs around the house.

#### Strong and weak relationships

When interpreting the social network aspects in terms of strong and weak relationships, we considered network members as strong relationships who were family, friends, members living in the same household, and those providing emotional support, as these usually reflect people who are emotionally close and with whom there can be recurrent interactions. Strong relationships also include members who live within walking distance or members from whom practical support was received, as this usually reflects people who are geographically close and with whom there has been in-person contact. We considered as weak relationships, network members who were acquaintances, and other (non-friend, non-family) relations, those who lived far away, and those who provided informational support, as according to Granovetter [18], new information is often obtained from weak ties.

### Statistical analyses

Participants with missing values on any of the outcome or other variables (n = 16) were excluded in analyses. Ordinal logistic regression analyses were performed for the count variable of preventive behaviors as outcome and the network characteristics as independent variables. Binary logistic regression analyses were performed for the four preventive behaviors (four outcomes) separately, with the network characteristics as independent variables, to gain more detailed insight into the specific behaviors. Multivariate logistic (for the separate preventive behaviors) and multivariate ordinal (for the count variable on behaviors) regression analyses were performed, calculating odds ratios (OR) with 95% confidence intervals. Multivariate model (I) included as independent variables the social network characteristics, and confounders (see below). Then, a multivariate model (II) was created including all social network aspects that were associated with the outcome (by a *p*-value <0.05) in model I, and including confounders. To obtain the final model (II), we used a stepwise backward method for the social network variables (while keeping the confounders in the model) [25]. Confounding variables were sociodemographic factors (sex, age, educational level) [26–29], chronic conditions (depression, type II diabetes mellitus, cardiovascular diseases, asthma/COPD) [24, 30–35], lifestyle factors (BMI, current smoking) [5, 36] and self-reported upper and lower respiratory infections in the previous two months [25, 37], as these variables previously were found to be associated with the dependent and independent variables. A priori, collinearity between network aspects was checked and ruled out (all correlations <0.66). A *p*-value < 0.05 was considered statistically significant. All analyses were performed using IBM SPSS Statistics (version 26.0).

## Results

### Study population

5,128 participants were included in analyses (Table 1 and S1 Fig). Table 1 presents the population characteristics.

### Current infection preventive behaviors

Most, i.e., 94%, of participants reported to wash their hands regularly with water and soap, 55% used paper tissues only, 19% touched their mouth, nose, and eyes as little as possible, and 39% kept physical distance from people who had symptoms of a respiratory infection. Of these four behaviors, the median count number was 2 (Table 1).

### Social network composition

The mean network size was 11 (Table 2). Overall, 46% of network members were family, 27% were friends, 58% were of the same sex and 40% were of the same (five years range) age as the respondent. A total of 15% network members lived in the same household, and 28% lived within walking distance. Respondents had contact in the past two weeks with a mean of ten people, i.e., with two network members contact was both in-person and by phone/internet, with six contact was exclusively in-person, and with two contact was exclusively by phone/internet; one network member was not contacted recently. Of respondents, 80% reported that their friends knew their family and 54% were member of a club. On average respondents received informational support from four, emotional support from six, and practical support from two network members.

**Table 2:** Social network characteristics of the SaNAE study population (n = 5,128).

Social network characteristics	% (n) or mean (sd)
**Structural characteristics of social networks**	
**Network size [range 0–40], i.e., the number of network members–mean(sd)**	11.3 (8.0)
**Type of relationship–mean (sd)**	
Proportion of network members who are family	46.3 (24.5)
Proportion of network members who are friends	26.6 (18.6)
Proportion of network members who are acquaintances	17.6 (15.5)
Proportion of network members who are extra members	8.1 (13.3)
**Homophily by sex–mean (sd)**	
Proportion of network members of the same sex	57.9 (22.1)
**Homophily by age–mean (sd)**	
Proportion of network members of the same age (~5 years)	39.7 (25.1)
Proportion of network members of younger age (<5 years)	41.9 (26.5)
Proportion of network members of older age (> 5 years)	17.0 (19.7)
**Proximity–mean (sd)**	
Proportion of network members who are household-members	15.3 (19.7)
Proportion of network members who live within walking distance	27.8 (24.4)
Proportion of network members who live less than 30 minutes away	36.4 (26.1)
Proportion of network members who live more than 30 minutes away	15.2 (19.3)
Proportion of network members who live further away	3.9 (10.7)
**Mode of contact–mean (sd) in past 2 weeks**	
Number of network members with whom respondents had both physical and phone/ internet contact*	1.9 (3.7)
Physical and phone/internet contact with 0 network members (%,n)	63.4 (3252)
Physical and phone/internet contact with 1–2 network members (%,n)	11.1 (570)
Physical and phone/internet contact with 3–5 network members (%,n)	12.1 (618)
Physical and phone/internet contact with 6–40 network members (%,n)	13.4 (688)
Number of network members with whom respondents had exclusively physical contact*	6.1 (5.6)
Exclusively physical contact with 0 network members (%,n)	9.8 (502)
Exclusively physical contact with 1–2 network members (%,n)	30.9 (1587)
Exclusively physical contact with 3–5 network members (%,n)	32.9 (1689)
Exclusively physical contact with 6–40 network members (%,n)	26.3 (1350)
Number of network members with whom respondents had exclusively phone/ internet contact*	2.1 (2.7)
Exclusively phone/internet contact with 0 network members (%,n)	36.5 (1874)
Exclusively phone/internet contact with 1–2 network members (%,n)	31.9 (1635)
Exclusively phone/internet contact with 3–5 network members (%,n)	21.6 (1107)
Exclusively phone/internet contact with 6–40 network members (%,n)	10.0 (512)
Number of network members with whom respondents had no recent contact	1.1 (2.2)
**Density–agree that most of the respondents’ friends know the respondents’ family**	80.5 (4129)
**Social participation–club membership**	54.0 (2769)
Sports club	34.0 (1746)
Talking group, internet group or self-help group	10.1 (520)
Charity organization	24.1 (1237)
Other (including religious groups)	31.4 (1608)
**Functional characteristics of social networks–mean, sd**	
**Number of network members from whom informational support was received**	4.2 (4.3)
**Number of network members from whom emotional support was received**	6.1 (5.8)
**Number of network members from whom practical support was received**	2.2 (2.4)

* Including respondents who reported zero network members for this mode of contact

### Network aspects associated with preventive behaviors

#### Count of (four) preventive behaviors

Adjusting for a range of potential confounders, in model I, positively associated structural social network characteristics were a larger network size, network members with older age than the respondent, each higher percent point in proportion friends, each higher percent point in proportion network members living far way, and a larger number of network members (>5 members versus 0) with whom there had been recent contact by phone/internet (and no contact in-person) (Table 3). All three functional network aspects were found positively associated, i.e., the likelihood of more preventive behaviors practiced increased with each additional network member giving practical support, emotional support, and informational support. Inversely associated were two structural network aspects, being the percent point increase of the proportion same-aged network members, and having a club membership, specifically a sports club membership and a charity organization membership.

**Table 3:** Multivariate model I—associations between social network characteristics and preventive behaviors in SaNAE study population.

	Preventive behavior for respiratory infections				
	Count preventive behaviors	Wash hands with water & soap	Use paper tissues	Touch face as little as possible	Keep distance from people with respiratory infectious disease symptoms
	OR (95% CI)	OR (95% CI)	OR (95% CI)	OR (95% CI)	OR (95% CI)
**Structural characteristics**					
**Network size**	**1.01 (1.00–1.02)** *	**1.03 (1.01–1.05)** ***	1.01 (1.00–1.01)	1.00 (1.00–1.01)	1.01 (1.00–1.01)
**Type of relationship**					
Proportion network members who are family members	0.89 (0.72–1.10)	0.85 (0.54–1.34)	0.93 (0.73–1.19)	1.01 (0.75–1.36)	0.86 (0.68–1.08)
Proportion network members who are friends	**1.32 (1.01–1.74)** *	1.04 (0.56–1.90)	1.28 (0.93–1.77)	1.25 (0.85–1.85)	1.19 (0.87–1.62)
Proportion network members who are acquaintances	0.86 (0.62–1.20)	1.35 (0.65–2.83)	0.98 (0.66–1.43)	0.76 (0.48–1.23)	0.85 (0.59–1.24)
Proportion network members who are other members	1.07 (0.73–1.56)	1.28 (0.54–3.06)	1.01 (0.65–1.58)	0.79 (0.46–1.37)	1.20 (0.79–1.84)
**Contacted children < five years of age**	1.01 (0.91–1.11)	1.16 (0.92–1.45)	**1.17 (1.03–1.32)** *	1.00 (0.87–1.16)	**0.87 (0.78–0.98)** *
**Living alone**	1.11 (0.98–1.26)	0.92 (0.70–1.21)	**1.20 (1.04–1.40)** *	1.01 (0.84–1.20)	1.06 (0.92–1.22)
**Homophily by sex**					
Proportion members of the same sex	0.79 (0.62–1.01) ^#^	1.17 (0.69–1.99)	0.90 (0.68–1.20)	0.80 (0.56–1.12)	0.77 (0.59–1.02) ^#^
**Homophily by age**					
Proportion members of same age	**0.73 (0.59–0.90)** **	**0.56 (0.36–0.88)** *	0.87 (0.68–1.11)	**0.67 (0.50–0.90)** **	0.82 (0.65–1.04)
Proportion members of younger age	1.18 (0.95–1.45)	1.39 (0.87–2.23)	**1.37 (1.07–1.76)** **	1.16 (0.86–1.57)	0.89 (0.70–1.13)
Proportion members of older age	**1.34 (1.02–1.77)** *	1.88 (0.96–3.68) ^#^	0.83 (0.60–1.15)	1.46 (1.00–2.13) ^#^	**1.55 (1.13–2.11)** **
**Proximity**					
Proportion members in house	0.94 (0.72–1.22)	0.77 (0.45–1.33)	0.96 (0.71–1.30)	0.98 (0.67–1.43)	0.96 (0.71–1.29)
Proportion members walking distance	0.87 (0.71–1.07)	0.75 (0.48–1.20)	0.85 (0.67–1.09)	1.08 (0.80–1.44)	0.86 (0.67–1.08)
Proportion members <30 minutes away	1.11 (0.91–1.34)	1.55 (1.00–2.39) ^#^	1.24 (0.99–1.56) ^#^	1.07 (0.81–1.41)	0.95 (0.77–1.19)
Proportion members >30 minutes away	0.87 (0.67–1.13)	0.80 (0.45–1.42)	0.85 (0.62–1.16)	**0.66 (0.45–0.98)** *	1.05 (.078–1.42)
Proportion members further away (far away)	**2.18 (1.37–3.51)** **	2.53 (0.74–8.67)	1.66 (0.95–2.91) ^#^	1.39 (0.72–2.68)	**2.02 (1.19–3.44)** **
**Mode of contact–in the last two weeks**					
Physical and phone/internet contact with:					
0 network members	ref	ref	ref	ref	ref
1–2 network members	0.88 (0.75–1.04)	0.97 (0.68–1.39)	0.84^#^ (0.69–1.01)	0.98 (0.78–1.24)	0.91 (0.75–1.10)
3–5 network members	0.95 (0.81–1.12)	1.06 (0.4–1.53)	0.95 (0.79–1.15)	0.81 (0.64–1.03) ^#^	0.96 (0.80–1.15)
6–40 network members	1.11 (0.95–1.29)	0.97 (0.69–1.37)	1.14 (0.94–1.37)	1.06 (0.86–1.31)	1.07 (0.90–1.27)
Exclusively physical contact with:					
0 network members	ref	ref	ref	ref	ref
1–2 network members	1.06 (0.88–1.28)	**1.54 (1.07–2.23)** *	1.07 (0.86–1.33)	1.11 (0.84–1.45)	0.93 (0.76–1.15)
3–5 network members	0.94 (0.78–1.13)	**1.49 (1.04–2.15)** *	0.99 (0.80–1.24)	1.03 (0.79–1.35)	0.82 (0.66–1.01) ^#^
6–40 network members	1.04 (0.86–1.26)	**1.78 (1.20–2.63)** **	1.01 (0.81–1.27)	1.10 (0.83–1.44)	0.95 (0.76–1.17)
Exclusively phone/internet contact with:					
0 network members	ref	ref	ref	ref	ref
1–2 network members	1.09 (0.97–1.24)	1.22 (0.93–1.59)	1.02 (0.89–1.18)	1.01 (0.85–1.21)	1.11 (0.97–1.28)
3–5 network members	1.14 (1.01–1.31) ^#^	**1.39 (1.01–1.92)** *	1.03 (0.87–1.21)	1.10 (0.90–1.34)	1.13 (0.97–1.33)
6–40 network members	**1.62 (1.35–1.95)** ***	**1.86 (1.15–3.00)** *	**1.36 (1.09–1.70)** **	**1.48 (1.16–1.89)** **	**1.45 (1.18–1.78)** ***
**Density (friends know family)**	0.95 (0.83–1.08)	1.19 (0.91–1.56)	0.97 (0.83–1.13)	1.04 (0.86–1.24)	0.89 (0.77–1.02)
**Social participation (any membership)**	**0.84 (0.76–0.93)** **	0.99 (0.79–1.25)	**0.85 (0.75–0.96)** **	**0.83 (0.72–0.96)** *	0.91 (0.81–1.02)
Membership sports club	**0.89 (0.80–1.00)** *	1.05 (0.82–1.34)	0.94 (0.83–1.07)	0.87 (0.75–1.02) ^#^	0.89 (0.79–1.10) ^#^
Membership internet, talking, self-help group	1.11 (0.94–1.31)	0.99 (0.68–1.45)	1.01 (0.83–1.23)	0.99 (0.78–1.25)	1.19 (0.98–1.43) ^#^
Membership charity	**0.88 (0.78–0.99)** *	1.06 (0.81–1.39)	**0.80 (0.69–0.92)** **	0.94 (0.80–1.11)	0.95 (0.83–1.09)
Membership other (including religious groups)	0.92 (0.83–1.03)	1.00 (.78–1.28)	0.89 (0.78–1.01) ^#^	0.98 (0.84–1.15)	0.96 (0.84–1.08)
**Functional characteristics**					
**Emotional support**	**1.01 (1.00–1.02)** *	**1.02 (1.00–1.05)** *	1.01 (1.00–1.02) ^#^	1.01 (0.99–1.02)	1.01 (1.00–1.02)
**Informational support**	**1.03 (1.02–1.04)** **	**1.05 (1.02–1.09)** **	**1.02 (1.01–1.04)** **	**1.03 (1.01–1.05)** ***	**1.02 (1.01–1.03)** **
**Practical support**	**1.02 (1.00–1.04)** *	1.02 (0.97–1.07)	1.01 (0.99–1.04)	**1.03 (1.00–1.06)** *	1.02 (1.00–1.05)

All analyses were adjusted for age, sex, educational level, self-reported upper & lower respiratory infections, type II diabetes mellitus, cardiovascular diseases, asthma/COPD, depression, BMI, and smoking. OR odds ratio, 95% CI; 95% confidence interval,

^#^ *p*<0.10,

* *p*<0.05,

**p<0.01,

***p<0.001.

In model II, assessing social network aspects independent from each other and adjusting for potential confounders, a set of factors remained independently associated with more infection-preventive behaviors practiced (Table 4). These factors were the structural social network aspects that indicated strong relationships, i.e., each higher percent point in the share of friends among network members, and the structural and functional aspects that indicated weak relationships, i.e., a larger proportion of network members living far away, a high number of network members (6–40 members) with whom there had been recent contact by phone/internet and no contact in-person, and each additional network member from whom informational support was received. Furthermore, inversely associated were the percent point increase of the proportion same-aged network members and having a club membership. A summary of the found associations for the count of preventive behaviors and for the behaviors separately is shown in S2 Table.

**Table 4:** Multivariate model II–independent associations between social network characteristics and preventive behaviors in SaNAE study population, adjusted for confounders.

	Preventive behaviors for respiratory infections				
	Count preventive behaviors	Wash hands with water & soap	Use paper tissues	Touch face as little as possible	Keep distance from people with respiratory infectious disease symptoms
	OR (95% CI)	OR (95% CI)	OR (95% CI)	OR (95% CI)	OR (95% CI)
**Structural characteristics**					
**Network size**		1.03 (1.01–1.05)***			
**Type of relationship**					
Proportion network members who are friends	1.34 (1.11–1.78)*				
**Contacted children < five years of age**			1.17 (1.03–1.32)*		
**Living alone**			1.24 (1.06–1.44)**		
**Homophily by age**					
Proportion members of same age	0.72 (0.58–0.89)**	0.57 (0.37–0.88)*		0.66 (0.49–0.90)**	
Proportion members of younger age			1.35 (1.05–1.74)*		
**Proximity**					
Proportion members living far away	1.82 (1.12–2.96)*				
**Mode of contact–in the last two weeks**					
Network members with whom respondents had exclusively phone or internet contact					
0 network members	Ref			Ref	Ref
1–2 network members	1.04 (0.92–1.18)			1.00 (0.84–1.20)	1.00 (0.84–1.19)
3–5 network members	1.06 (0.91–1.22)			1.06 (0.87–1.30)	1.05 (0.86–1.29)
6–40 network members	1.41 (1.16–1.71)**			1.35 (1.04–1.74)*	1.33 (1.03–1.72)*
**Social participation (club membership)**	0.82 (0.74–0.91)***			0.82 (0.71–0.95)**	
Membership charity			0.77 (0.67–0.89)***		
**Functional characteristics**					
**Informational support**	1.02 (1.01–1.04)***		1.02 (1.01–1.04)**	1.03 (1.01–1.04)**	1.02 (1.01–1.04)**

All statistically significant variables found in model I were added in model II, and then b-step procedure was applied. All analyses were adjusted for age, sex, educational level, type II diabetes mellitus, cardiovascular diseases, asthma/COPD, depression, BMI, smoking and upper & lower respiratory infections. OR odds ratio, 95% CI; 95% confidence interval.

* *p*<0.05,

**p<0.01,

***p<0.001.

#### Washing hands regularly with water and soap

In model I, several associated social network aspects were similar to those found associated with the count of preventive behaviors (Table 3), i.e., positively associated were larger network size, more network members with whom there was exclusively phone/internet contact, emotional support and informational support, and inversely associated were proportion network members of the same age. In addition, more network members with whom there was exclusively physical contact was associated with washing hands regularly.

In model II, overall network size (positively) and proportion network members of the same age (inversely) remained independently associated with washing hands regularly (Table 4).

#### Using only paper (not cloth) handkerchiefs/tissues

In model I, several associated social network aspects were similar to those associated with the count of preventive behaviors (Table 3), i.e., positively associated were contact with more network members exclusively via phone/internet, receiving informational support from more network members, and inversely associated were having a club membership, specifically, charity club. In addition, in model I, positively associated structural social network aspects associated with using paper tissues only were: having contact with children under the age of five, living alone, and a larger proportion network members of a younger age.

In model II, contact with children under the age of five, living alone, a larger proportion network members of younger age, and receiving informational support, remained independently positively associated; charity club membership remained inversely associated (Table 4).

#### Touch face as little as possible

In model I, several associated social network aspects were similar to those found associated with the count of preventive behaviors (Table 3), i.e., positively associated were more network members with whom there was exclusively phone/internet contact and receiving practical or informational support from more members, and inversely associated were a larger proportion members of the same age and having a club membership. In addition, inversely associated in model I was a larger proportion members living more than 30 minutes away.

In model II, network members with whom there was exclusively phone/internet contact and informational support from more members remained independently positively associated, and a larger proportion members of the same age and having a club membership remained inversely associated (Table 4).

#### Keep distance from people who have symptoms of respiratory infectious disease

In model I, several associated social network aspects were similar to those found associated with the count of preventive behaviors (Table 3), i.e., positively associated were larger proportions of network members who were older than the respondent, larger proportions of network members living far away, more network members with whom there only was exclusively contact by phone or internet, and received informational support from more network members. In addition in model I, inversely associated with keeping distance from people who have symptoms was having contact with children under the age of five.

In model II, more network members with whom there only was exclusively contact by phone/internet, and received informational support remained independently positively associated (Table 4).

## Discussion

The SaNAE-study aimed to assess in middle aged and older people living independently in the community, which aspects of their social networks related to the practice of infection preventive behaviors. This is the first study that investigated a broad range of structural and functional social networks aspects, by different types, strengths, and modes of interactions, and their independent effects on infection prevention behavior. Data were collected just before the COVID-19 pandemic further magnified the urgency for effective infection-prevention.

The main finding (model II) was that the likelihood of practicing more preventive behaviors was independently increased by network aspects that reflected ‘weak relationships’. These comprised structural aspects, i.e., when a person’s social network included a higher share of geographically distant living people and when the network included many network members with whom there was online/telephone contact only (and no in-person contact). Weak relationships also comprised a functional network aspect, i.e., having more network members who provide informational support. Weak ties are assumed to provide access to novel information, such as information that enable respondents to adopt and maintain infection-preventive behaviors [16].

The likelihood of practicing preventive behaviors was also increased by a structural social network aspect reflecting ‘strong ties’, i.e., when a person’s social network included a higher share of friends. Friends are likely to indirectly control health behavior, e.g., act as behavioral role models, introducing descriptive norms for specific behaviors [14, 19, 20, 38]. Friends may also provide all types of social support, i.e., emotional, practical as well as informational. Although no specific ‘friends-provided’ support type could be identified in our data, the association with share of friends was independent from receiving informational support, suggesting that the ‘effect’ of friends goes beyond providing informational support [17, 18]. Except for friends, no other social network aspect that may indicate ‘strong relations’ was found to be associated, as family members or neighbors, who may mainly provide emotional and practical support. Close family, spouses, or household members, have previously been identified important in promoting other lifestyle behaviors as healthy eating, smoking cessation, and vaccination uptake [3, 5–7]. It should be noted that what appears important from our model II, is to have a higher share of friends in one’s total network composition, not just having more friends.

Further, we noted two social network aspects that decreased the likelihood of practicing more preventive behaviors. These were age-homophily, i.e., when a larger share of the existing network was composed by same-age members, and social participation, i.e., having a club membership. Whether these aspects indicated strong or weak relations was unknown, as not all network aspects could be attributed to relationship-strength. One of the possible mechanisms explaining these inverse associations may be social influence. Individuals tend to adapt their behaviors or attitudes as a result of interaction with others [13, 39]. Another mechanism could be social selection processes. People tend to connect with other individuals who are similar to themselves [11, 40, 41].

In this study, we were primarily interested in the count of the four behaviors, rather than the separate behaviors alone, because the more behaviors are practiced, the greater the potential reduction in risk to acquire respiratory infections. Nevertheless, by also exploring the separate behaviors, we noted some additional social network aspects to be associated in model II, i.e., a larger overall network size in washing hands (thus in this behavior the mode of contact and support received was less important than the absolute number of relationships). For using paper tissues, having contact with children younger than five years of age, the proportion of network members of a younger age, and living alone was found to be associated (alongside informational support).

The explanation of the associations of social network aspects is driven by solid and assumed most likely theoretical explanatory frameworks, as outlined before in the introduction. Alternative explanations of observed associations are also possible. For example, the association with online/telephone only interactions may also reflect an active refraining from in-person contacts due to a fear of getting infected or due to fear of transmitting an infection to a network member, or when not being able/not wanting to have in-person contact due to illness or infection symptoms in the respondent. While the first possibility (fear) was not measured in our study, the latter two were considered unlikely to explain our results, since both multivariate models were adjusted for respondents’ self-reported respiratory infections.

Social networks have the potential to increase the effectiveness and reach of preventive strategies to promote various health behaviors. This study identified several social network aspects that were positively associated with behaviors known to be effective in preventing respiratory infections. Results indicate that weak relationships that provide informational support, with whom there can be online/telephone interactions, and strong relationships, i.e., to have a large share of friends in the network, are potentially likely to play an important role in the adoption of infection prevention behaviors. It may now be further explored how to employ these network aspects for the strengthening of non-pharmaceutical infection-control strategies in the middle aged and older population, which is especially relevant in the current context of the COVID-19 pandemic. Basic preventive behavior can be induced by role models (e.g., friends) or receiving advice or information from more distant network members. Further, we may invent new strategies to increase the share of friends in the composition of one’s network and employ such connections to promote infection preventive behaviors. Specifically, the strengthening of the so called ‘weak relationships’, as the modes of interactions that provide informational support, that do not have to be in-person, thus possibly via online channels, is worth to be explored further. Such online contact opportunities may especially be useful in the current situation, where the COVID-19 pandemic led to widespread application of isolation, quarantine, and physical distancing, already likely shifting the use of these modes of interactions. Previous studies have shown that the internet can be used to receive social support from or social interaction with others [42, 43]. This reasoning leads to exploring the promotion of new/linking to already existing online, interactive, informal information platforms that allow people to exchange information and advice. Existing public health websites may be included because these provide information about the reasons why preventive behavior can be important and how to apply preventive measures; interacting with social network members contributes to increasing awareness of one’s behavior. Health care organizations could play an important role in such platforms, as they can facilitate validated information [43]. Future studies need to investigate how to promote such interactive, online platforms.

A strength of this study is the use of a name generator questionnaire to inquire a person’s social network. The use of a name generator questionnaire is known to be practical and reliable [44]. Also, a very large study population with only few missing data was used for this study, yielding substantial study power to examine a broad range of variables.

There are also limitations. (1) Although by our measures we assumed that the social network aspects precede the infection-preventive behaviors (current/recent behaviors are reported), the nature of the study is cross-sectional, precluding conclusions on the causality of observed effects. Yet, it is likely that the social network composition, that is usually more or less stable at least for some time in non-pandemic times [45], precedes the current reported infection prevention behaviors. (2) Strong and weak relationships were defined by rough approximation only (more detailed information on relationship-strength was not available in the dataset). (3) Our outcome measures were behaviors, which were self-reported and therefore subject to information bias. Participants were asked if they washed their hands regularly with water and soap, but regularly was not further defined. Self-reporting also might result in socially desirable answers and thus possibly an overestimation of preventive behaviors. However, the questionnaire was online and could be filled in in the private environment of one’s own home, limiting social desirability bias. Also, handwashing was reported quite frequently, somewhat limiting the power to detect associations when evaluating this specific outcome. (4) Respondents represented a middle aged to older population living independently at home, of whom one third reported one or more chronic conditions. Nearly half of the respondents were highly educated. Thus overall, respondents in this study may not completely represent the adult population of Limburg, the Dutch region of study.

## Conclusion

This study revealed which social relations are likely important in the adoption of infection-preventive behaviors. ‘Weak relations’ as more geographically distant network members, and those who provide informational support, and a larger share of ‘strong relations’ i.e., friends, may play a role and employment of these network connections now could be explored to be employed to strengthen infectious diseases control programs.

## Supporting information

S1 Fig Flowchart of the SaNAE study population. (TIF)

S1 Table Non-response analysis between respondents and non-respondents, using Chi square tests and an independent samples t-test. (DOCX)

S2 Table Summary associations between social network characteristics and prevention behavior. (DOCX)

S3 Table Univariate logistic and ordinal regression analyses–associations between social network characteristics and infection prevention behaviors. (DOCX)
