# Supplementary figures and images for: Social networks and infectious diseases prevention behavior: A cross-sectional study in people aged 40 years and older
Source: PLoS One. 2021 May 19;16(5):e0251862. doi: 10.1371/journal.pone.0251862 (PMC8133464; doi:10.1371/journal.pone.0251862)

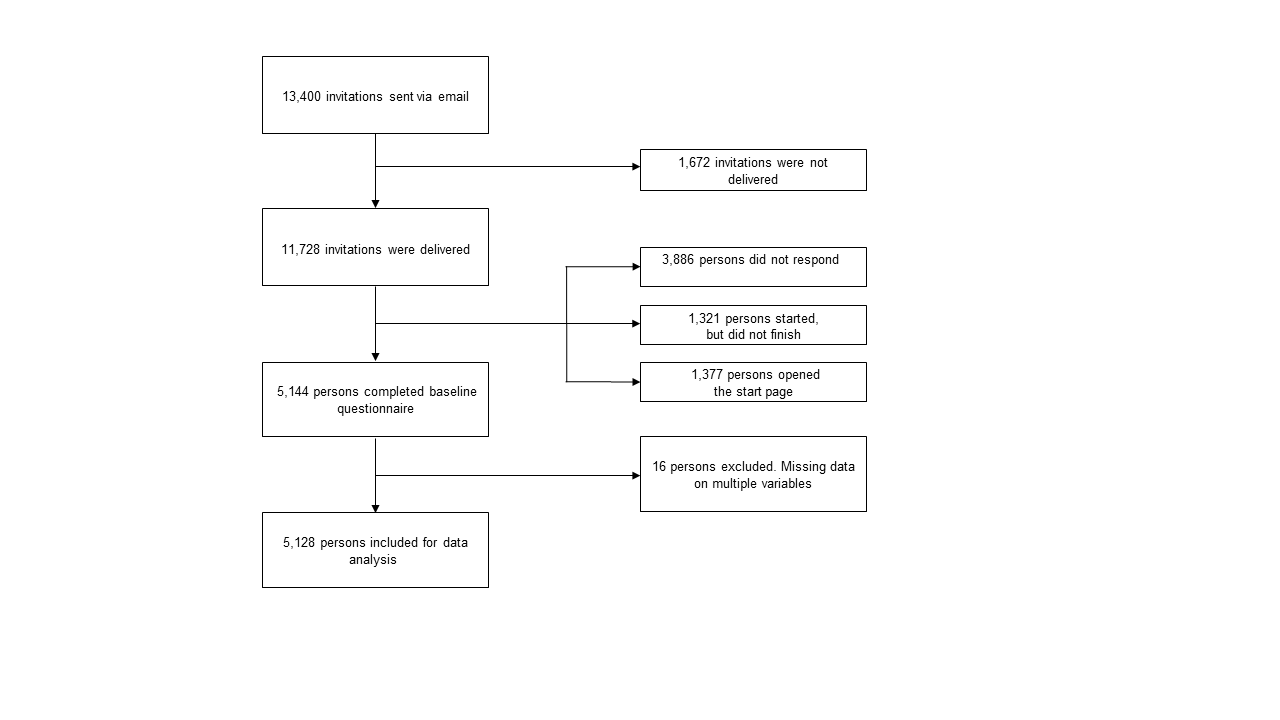

Supplement: S1 Fig — (TIF) [file pone.0251862.s001.tif]
