# Supplementary material for: Social networks and infectious diseases prevention behavior: A cross-sectional study in people aged 40 years and older
Source: PLoS One. 2021 May 19;16(5):e0251862. doi: 10.1371/journal.pone.0251862 (PMC8133464; doi:10.1371/journal.pone.0251862)
Supplement: S1 Table — (DOCX) [file pone.0251862.s002.docx]

**S1 Table. Non-response analysis between respondents and non-respondents, using Chi Square tests and an independent samples t-test.**

|  | All participants who were invited | All participants who completed SaNAE 2019° | Non-respondents° | p-value on difference between respondents and non-respondents |
| --- | --- | --- | --- | --- |
| Number of persons | 13484 | 5128 | 8223 | - |
| Sex, man | 55.1% (7428) | 54.4% (2789) | 55.6% (4635) | 0.172 |
| Age (mean, sd) | 64.0 (11.1) | 63.3 (10.3) | 64.4 (11.6) | < 0.001 |
| Educational level° |  |  |  | < 0.001 |
| Low | 29.7% (4011) | 23.1% (1187) | 33.8% (2820) |  |
| Medium | 33.9% (4566) | 35.4% (1813) | 33.0% (2752) |  |
| High | 35.5% (4784) | 41.5% (2128) | 31.8% (2651) |  |

**^°^** Missing data (n=117) among the non-respondents and missing data (n=16) among participants who completed SaNAE 2019.
