# Supplementary material for: Social networks and infectious diseases prevention behavior: A cross-sectional study in people aged 40 years and older
Source: PLoS One. 2021 May 19;16(5):e0251862. doi: 10.1371/journal.pone.0251862 (PMC8133464; doi:10.1371/journal.pone.0251862)
Supplement: S2 Table — (DOCX) [file pone.0251862.s003.docx]

**S2 Table. Summary of found associations between social network characteristics and preventive behaviors in model I and II**

|  | Count preventive behaviors | Wash hands with water & soap | Use paper tissues | Touch face as little as possible | Keep distance from people with respiratory symptoms |
| --- | --- | --- | --- | --- | --- |
| Positively associated |  |  |  |  |  |
| Structural characteristics | | | | | |
| Network size | *A* | A |  |  |  |
| Type of relationship |  |  |  |  |  |
| Proportion network members who are friends | A |  |  |  |  |
| Homophily by age |  |  |  |  |  |
| Proportion members of older age | *A* |  |  |  | *A* |
| Proportion members of younger age |  |  | **A** |  |  |
| Contact with children < five years of age |  |  | **A** |  |  |
| Living alone |  |  | **A** |  |  |
| Proximity |  |  |  |  |  |
| Proportion members further away (far away) | A |  |  |  | *A* |
| Mode of contact |  |  |  |  |  |
| Exclusively physical contact with more people |  | ***A*** |  |  |  |
| Exclusively phone/internet contact with 6-40 network members | A | *A* | A | A | A |
| Functional characteristics | | | | | |
| Emotional support | *A* | *A* |  |  |  |
| Informational support | A | *A* | A | A | A |
| Practical support | *A* |  |  | *A* | *A* |
|  |  |  |  |  |  |
| Inversely associated |  |  |  |  |  |
| Structural characteristics |  |  |  |  |  |
| Homophily by age |  |  |  |  |  |
| Proportion members of same age | I | I |  | I |  |
| Club membership | I |  | I | I | *I* |
| Proximity |  |  |  |  |  |
| Proportion members >30 minutes away |  |  |  | ***I*** |  |
| Contact with children |  |  |  |  | ***I*** |

Multivariate model I is italicized, model II is underlined. Network characteristic shown in bold are only found to be associated with the behaviors separately.

A Positively associated network characteristics.

I Inversely associated network characteristics**.**
