# Supplementary material for: Social networks and infectious diseases prevention behavior: A cross-sectional study in people aged 40 years and older
Source: PLoS One. 2021 May 19;16(5):e0251862. doi: 10.1371/journal.pone.0251862 (PMC8133464; doi:10.1371/journal.pone.0251862)
Supplement: S3 Table — (DOCX) [file pone.0251862.s004.docx]

**S3 Table. Unadjusted univariate logistic and ordinal regression analyses of social network characteristics and infection prevention behaviors.**

|  | Preventive behavior for respiratory infections | | | | |
| --- | --- | --- | --- | --- | --- |
|  | Count preventive behaviors | Wash hands with water & soap | Use paper tissues | Touch face as little as possible | Keep distance from people with respiratory symptoms |
|  | OR (95% CI) | OR (95% CI) | OR (95% CI) | OR (95% CI) | OR (95% CI) |
| Structural characteristics | | | | | |
| Network size | **1.02 (1.01-1.02) ^***^** | **1.04 (1.02-1.05) ^***^** | **1.02 (1.01-1.03) ^***^** | 1.01 (1.00-1.02) | 1.00 (1.00-1.01) |
| Type of relationship |  |  |  |  |  |
| Proportion network members who are family members | **0.73 (0.59-0.89) ^**^** | 0.78 (0.50-1.22) | **0.64 (0.51-0.80) ^***^** | 0.94 (0.71-1.26) | 0.89 (0.71-1.12) |
| Proportion network members who are friends | **1.78 (1.36-2.33) ^***^** | 1.18 (0.64-2.16) | **2.26 (1.67-3.05) ^***^** | 1.39 (0.96-2.02) ^#^ | 1.14 (0.85-1.55) |
| Proportion network members who are acquaintances | 0.99 (0.72-1.37) | 1.54 (0.74-3.23) | 1.40 (0.98-2.00) ^#^ | 0.75 (0.48-1.19) | 0.76 (0.53-1.09) |
| Proportion network members who are other members | 1.09 (0.75-1.59) | 1.32 (0.55-3.18) | 0.89 (0.59-1.35) | 0.88 (0.52-1.50) | 1.34 (0.88-2.04) |
| Contacted children < five years of age | 1.05 (0.95-1.15) | 1.19 (0.95-1.49) | **1.27 (1.14-1.42) ^***^** | 0.99 (0.86-1.14) | **0.84 (0.75-0.94) ^**^** |
| Living alone | **1.20 (1.06-1.35) ^**^** | 0.89 (0.68-1.17) | **1.21 (1.05-1.38) ^**^** | 1.10 (0.93-1.30) | **1.16 (1.01-1.33) ^*^** |
| Homophily by sex |  |  |  |  |  |
| Proportion members of the same sex | **1.60 (1.27-2.01) ^***^** | 1.45 (0.88-2.39) | **2.86 (2.22-3.69) ^***^** | 1.15 (0.84-1.59) | 0.79 (0.61-1.02) ^#^ |
| Homophily by age |  |  |  |  |  |
| Proportion members of same age | **0.71 (0.58-0.87) ^**^** | **0.58 (0.37-0.90) ^*^** | 1.04 (0.83-1.29) | **0.60 (0.45-0.80) ^***^** | **0.69 (0.55-0.86) ^**^** |
| Proportion members of younger age | 1.06 (0.88-1.28) | 1.25 (0.81-1.91) | **0.68 (0.55-0.83) ^***^** | **1.36 (1.04-1.77) ^*^** | **1.37 (1.11-1.69) ^**^** |
| Proportion members of older age | **1.65 (1.28-2.12) ^***^** | **1.99 (1.07-3.72) ^*^** | **2.31 (1.73-3.08) ^***^** | 1.26 (0.89-1.78) | 0.94 (0.71-1.25) |
| Proximity |  |  |  |  |  |
| Proportion members in house | **0.63 (0.49-0.81) ^***^** | 0.72 (0.42-1.22) | **0.65 (0.49-0.86) ^**^** | 0.71 (0.49-1.04) ^#^ | 0.76 (0.57-1.02) ^#^ |
| Proportion members walking distance | 1.02 (0.83-1.25) | 0.81 (0.51-1.27) | 0.90 (0.71-1.12) | 1.31 (0.99-1.74) ^#^ | 1.02 (0.81-1.29) |
| Proportion members <30 minutes away | **1.23 (1.02-1.49) ^*^** | **1.62 (1.05-2.51) ^*^** | **1.57 (1.28-1.94) ^***^** | 1.08 (0.83-1.41) | 0.88 (0.71-1.09) |
| Proportion members >30 minutes away | 0.92 (0.71-1.19) | 0.81 (0.46-1.42) | 0.92 (0.69-1.22) | **0.68 (0.47-0.99) ^*^** | 1.11 (0.83-1.48) |
| Proportion members further away (far away) | **2.08 (1.30-3.32) ^**^** | 2.27 (0.67-7.78) | 1.31 (0.78-2.21) | 1.38 (0.73-2.60) | **2.43 (1.44-4.08) ^**^** |
| Type of contact – in the last two weeks |  |  |  |  |  |
| Physical and phone/internet contact with: |  |  |  |  |  |
| 0 network members | Ref | Ref | Ref | Ref | Ref |
| 1-2 network members | 0.88 (0.75-1.03) | 0.95 (0.67-1.36) | 0.87 (0.73-1.04) | 0.97 (0.77-1.22) | 0.91 (0.76-1.09) |
| 3-5 network members | 0.95 (0.81-1.11) | 1.07 (0.74-1.53) | 1.05 (0.89-1.25) | **0.78 (0.62-0.99) ^*^** | 0.90 (0.75-1.07) |
| 6-40 network members | **1.20 (1.04-1.40) ^*^** | 1.02 (0.73-1.43) | **1.49 (1.26-1.76) ^***^** | 1.03 (0.84-1.27) | 0.96 (0.81-1.14) |
| Exclusively physical contact with: |  |  |  |  |  |
| 0 network members | Ref | ref | Ref | Ref | Ref |
| 1-2 network members | 1.06 (0.88-1.27) | **1.52 (1.06-2.18) ^*^** | 0.93 (0.76-1.14) | 1.16 (0.89-1.51) | 1.04 (0.85-1.28) |
| 3-5 network members | 1.01 (0.84-1.21) | **1.53 (1.07-2.19) ^*^** | 0.96 (0.79-1.18) | 1.13 (0.86-1.46) | 0.92 (0.75-1.13) |
| 6-40 network members | 1.14 (0.95-1.38) | **1.89 (1.29-2.78) ^**^** | 1.07 (0.87-1.31) | 1.19 (0.91-1.55) | 1.02 (0.82-1.25) |
| Exclusively phone/internet contact with: |  |  |  |  |  |
| 0 network members | Ref | Ref | Ref | Ref | Ref |
| 1-2 network members | **1.19 (1.06-1.35) ^**^** | 1.23 (0.95-1.60) | **1.18 (1.03-1.35) ^*^** | 1.06 (0.89-1.26) | 1.13 (0.98-1.29) ^#^ |
| 3-5 network members | **1.38 (1.20-1.57) ^***^** | **1.43 (1.05-1.95) ^*^** | **1.39 (1.19-1.61) ^***^** | **1.22 (1.01-1.48) ^*^** | **1.18 (1.01-1.37) ^*^** |
| 6-40 network members | **2.00 (1.67-2.39) ^***^** | **1.92 (1.20-3.06) ^**^** | **1.88 (1.53-2.31) ^***^** | **1.63 (1.29-2.06) ^***^** | **1.49 (1.22-1.82) ^***^** |
| Density (friends know family) | 0.96 (0.85-1.09) | 1.25 (0.96-1.64) ^#^ | 0.99 (0.86-1.14) | 1.05 (0.88-1.25) | 0.89 (0.77-1.02) |
| Social participation (any membership) | **0.87 (0.79-0.96) ^**^** | 1.02 (0.82-1.28) | **0.88 (0.78-0.98) ^*^** | **0.85 (0.74-0.98) ^*^** | 0.93 (0.83-1.04) |
| Membership sports club | 0.92 (0.83-1.02) | 1.09 (0.86-1.38) | 1.01 (0.90-1.13) | 0.89 (0.76-1.03) | **0.87 (0.78-0.99) ^*^** |
| Membership internet, talking, self-help group | 1.11 (0.94-1.31) | 1.00 (0.69-1.45) | 0.99 (0.82-1.18) | 1.00 (0.79-1.26) | **1.25 (1.04-1.51) ^*^** |
| Membership charity | 0.91 (0.81-1.03) | 1.08 (0.83-1.42) | **0.80 (0.70-0.91) ^**^** | 0.99 (0.84-1.16) | 1.02 (0.90-1.16) |
| Membership other (including religious groups | 0.94 (0.85-1.05) | 1.01 (0.80-1.29) | **0.85 (0.76-0.96) ^**^** | 1.02 (0.88-1.18) | 1.04 (0.92-1.17) |
| Functional characteristics | | | | | |
| Emotional support | **1.02 (1.01-1.03) ^***^** | **1.03 (1.01-1.05) ^*^** | **1.03 (1.02-1.04) ^***^** | 1.01 (1.00-1.02) | 1.00 (0.99-1.01) |
| Informational support | **1.04 (1.03-1.05) ^***^** | **1.06 (1.02-1.09) ^**^** | **1.05 (1.04-1.07) ^***^** | **1.03 (1.01-1.04) ^***^** | 1.01 (1.00-1.02) |
| Practical support | 1.02 (1.00-1.04) | 1.02 (0.97-1.07) | 1.01 (0.99-1.04) | 1.02 (0.99-1.05) | 1.01 (0.99-1.04) |

OR odds ratio, 95% CI; 95% confidence interval, ^#^ *p*<0.10, **^*^ *p*<0.05, ^**^*p*<0.01, ^***^*p*<0.001.**
