# Supplementary material for: Social networks and infectious diseases prevention behavior: A cross-sectional study in people aged 40 years and older
Source: PLoS One. 2021 May 19;16(5):e0251862. doi: 10.1371/journal.pone.0251862 (PMC8133464; doi:10.1371/journal.pone.0251862)
Supplement: Peer review history

# Peer review: Decision Letter 0

**DOI:** 10.1371/journal.pone.0251862.r001

**Authors:** Luigi Lavorgna

21 Apr 2021

PONE-D-21-06252

Social networks and infectious diseases prevention behavior: a cross-sectional study in people aged 40 years and older

PLOS ONE

Thank you for submitting your manuscript to PLOS ONE. After careful consideration, we feel that it has merit but does not fully meet PLOS ONE’s publication criteria as it currently stands. Therefore, we invite you to submit a revised version of the manuscript that addresses the points raised during the review process.

Please submit your revised manuscript by April 30th. If you will need more time than this to complete your revisions, please reply to this message or contact the journal office at plosone@plos.org. When you're ready to submit your revision, log on to https://www.editorialmanager.com/pone/ and select the 'Submissions Needing Revision' folder to locate your manuscript file.

Please include the following items when submitting your revised manuscript:

- A rebuttal letter that responds to each point raised by the academic editor and reviewer(s). You should upload this letter as a separate file labeled 'Response to Reviewers'.
- A marked-up copy of your manuscript that highlights changes made to the original version. You should upload this as a separate file labeled 'Revised Manuscript with Track Changes'.
- An unmarked version of your revised paper without tracked changes. You should upload this as a separate file labeled 'Manuscript'.

If you would like to make changes to your financial disclosure, please include your updated statement in your cover letter. Guidelines for resubmitting your figure files are available below the reviewer comments at the end of this letter.

If applicable, we recommend that you deposit your laboratory protocols in protocols.io to enhance the reproducibility of your results. Protocols.io assigns your protocol its own identifier (DOI) so that it can be cited independently in the future. For instructions see: http://journals.plos.org/plosone/s/submission-guidelines#loc-laboratory-protocols. Additionally, PLOS ONE offers an option for publishing peer-reviewed Lab Protocol articles, which describe protocols hosted on protocols.io. Read more information on sharing protocols at https://plos.org/protocols?utm_medium=editorial-email&utm_source=authorletters&utm_campaign=protocols.

We look forward to receiving your revised manuscript.

Kind regards,

Luigi Lavorgna

Academic Editor

PLOS ONE

Journal Requirements:

When submitting your revision, we need you to address these additional requirements.

1. Please ensure that your manuscript meets PLOS ONE's style requirements, including those for file naming. The PLOS ONE style templates can be found at

https://journals.plos.org/plosone/s/file?id=wjVg/PLOSOne_formatting_sample_main_body.pdf and

https://journals.plos.org/plosone/s/file?id=ba62/PLOSOne_formatting_sample_title_authors_affiliations.pdf

2. Please review your reference list to ensure that it is complete and correct. If you have cited papers that have been retracted, please include the rationale for doing so in the manuscript text, or remove these references and replace them with relevant current references. Any changes to the reference list should be mentioned in the rebuttal letter that accompanies your revised manuscript. If you need to cite a retracted article, indicate the article’s retracted status in the References list and also include a citation and full reference for the retraction notice.

3. Please provide additional details regarding participant consent. In the ethics statement in the Methods and online submission information, please ensure that you have specified (i) whether consent was informed and (ii) what type you obtained (for instance, written or verbal, and if verbal, how it was documented and witnessed). If your study included minors, state whether you obtained consent from parents or guardians. If the need for consent was waived by the ethics committee, please include this information.

If you are reporting a retrospective study of medical records or archived samples, please ensure that you have discussed whether all data were fully anonymized before you accessed them and/or whether the IRB or ethics committee waived the requirement for informed consent. If patients provided informed written consent to have data from their medical records used in research, please include this information.

4. We note that you have included the phrase “data not shown” in your manuscript. Unfortunately, this does not meet our data sharing requirements. PLOS does not permit references to inaccessible data. We require that authors provide all relevant data within the paper, Supporting Information files, or in an acceptable, public repository. Please add a citation to support this phrase or upload the data that corresponds with these findings to a stable repository (such as Figshare or Dryad) and provide and URLs, DOIs, or accession numbers that may be used to access these data. Or, if the data are not a core part of the research being presented in your study, we ask that you remove the phrase that refers to these data.

5. We note that you have indicated that data from this study are available upon request. PLOS only allows data to be available upon request if there are legal or ethical restrictions on sharing data publicly. For information on unacceptable data access restrictions, please see http://journals.plos.org/plosone/s/data-availability#loc-unacceptable-data-access-restrictions.

In your revised cover letter, please address the following prompts:

a) If there are ethical or legal restrictions on sharing a de-identified data set, please explain them in detail (e.g., data contain potentially identifying or sensitive patient information) and who has imposed them (e.g., an ethics committee). Please also provide contact information for a data access committee, ethics committee, or other institutional body to which data requests may be sent.

b) If there are no restrictions, please upload the minimal anonymized data set necessary to replicate your study findings as either Supporting Information files or to a stable, public repository and provide us with the relevant URLs, DOIs, or accession numbers. Please see http://www.bmj.com/content/340/bmj.c181.long for guidelines on how to de-identify and prepare clinical data for publication. For a list of acceptable repositories, please see http://journals.plos.org/plosone/s/data-availability#loc-recommended-repositories.

We will update your Data Availability statement on your behalf to reflect the information you provide.

[Note: HTML markup is below. Please do not edit.]

Reviewers' comments:

Reviewer's Responses to Questions

**Comments to the Author**

1. Is the manuscript technically sound, and do the data support the conclusions?

The manuscript must describe a technically sound piece of scientific research with data that supports the conclusions. Experiments must have been conducted rigorously, with appropriate controls, replication, and sample sizes. The conclusions must be drawn appropriately based on the data presented. 

Reviewer #1: Yes

Reviewer #2: Yes

**********

2. Has the statistical analysis been performed appropriately and rigorously? 

Reviewer #1: Yes

Reviewer #2: Yes

**********

3. Have the authors made all data underlying the findings in their manuscript fully available?

The PLOS Data policy requires authors to make all data underlying the findings described in their manuscript fully available without restriction, with rare exception (please refer to the Data Availability Statement in the manuscript PDF file). The data should be provided as part of the manuscript or its supporting information, or deposited to a public repository. For example, in addition to summary statistics, the data points behind means, medians and variance measures should be available. If there are restrictions on publicly sharing data—e.g. participant privacy or use of data from a third party—those must be specified.

Reviewer #1: Yes

Reviewer #2: Yes

**********

4. Is the manuscript presented in an intelligible fashion and written in standard English?

PLOS ONE does not copyedit accepted manuscripts, so the language in submitted articles must be clear, correct, and unambiguous. Any typographical or grammatical errors should be corrected at revision, so please note any specific errors here.

Reviewer #1: Yes

Reviewer #2: Yes

**********

5. Review Comments to the Author

Please use the space provided to explain your answers to the questions above. You may also include additional comments for the author, including concerns about dual publication, research ethics, or publication ethics. (Please upload your review as an attachment if it exceeds 20,000 characters)

Reviewer #1: In this study, Steijvers et al. explored the role of social networks in non-pharmaceutical infection preventive behaviours, especially for respiratory infectious diseases. By collecting data from a very large sample in the pre-COVID era, authors reported that to practice preventive behaviours was independently increased by social network aspects that reflected ‘weak relationships’. The study performed a through analysis of different social network aspects and I believe that the analysis is properly performed. Only few points need to be addressed before the paper can be suitable for publication.

• Introduction is long. It can be summarizes by deleting the “The composition of social networks can be […] among middle aged and older adults in the Netherlands” paragraph;

• Statistical analysis methods are not clear. I would kindly ask to rephrase the whole paragraph as to better define, for each model the dependent and independent variable for sake of clarity. Also, it is not clear to me why authors entered in a step-wise regression some variables resulting from the previous model, and other ‘confounding founders’. What are these factors? How you chose them?

• In table 2, I do not foresee any advantage in reporting different levels of p-value. As author stated in the statistical section that 0.05 is the statistical significance value they should only report significant vs non-significant values;

Reviewer #2: Interesting and innovative article showing how social networks potentially can increase the effectiveness and can promote healthy behaviors, taking advantage from social relationship and experience from peers. Social media can help people with health concerns or chronic disease, finding in Web communities a comforting source for information exchange, debate, and knowledge enrichment. I suggest the authors to implement Discussion with previous reports demonstrating this value (see Lavorgna L et al. Interact J Med Res. 2017) for health care organizations and beneficial repercussion reaching institutional stakeholders.

Resort to on-line survey has been widely amplified during pandemic due to need of reach a large population with a digital approach. Authors could emphasize the importance and usefulness of this easy and well accepted tool in the Conclusion.

**********

6. PLOS authors have the option to publish the peer review history of their article (what does this mean?). If published, this will include your full peer review and any attached files.

If you choose “no”, your identity will remain anonymous but your review may still be made public.

**Do you want your identity to be public for this peer review?** For information about this choice, including consent withdrawal, please see our Privacy Policy.

Reviewer #1: No

Reviewer #2: No

[NOTE: If reviewer comments were submitted as an attachment file, they will be attached to this email and accessible via the submission site. Please log into your account, locate the manuscript record, and check for the action link "View Attachments". If this link does not appear, there are no attachment files.]

While revising your submission, please upload your figure files to the Preflight Analysis and Conversion Engine (PACE) digital diagnostic tool, https://pacev2.apexcovantage.com/. PACE helps ensure that figures meet PLOS requirements. To use PACE, you must first register as a user. Registration is free. Then, login and navigate to the UPLOAD tab, where you will find detailed instructions on how to use the tool. If you encounter any issues or have any questions when using PACE, please email PLOS at figures@plos.org. Please note that Supporting Information files do not need this step.

# Author comment: Author response to Decision Letter 0

**DOI:** 10.1371/journal.pone.0251862.r002

29 Apr 2021

We thank the editor and the reviewers for their constructive and positive comments, all addressed below in our point-by-point reply.

1. Please ensure that your manuscript meets PLOS ONE's style requirements, including those for file naming. The PLOS ONE style templates can be found at https://journals.plos.org/plosone/s/file?id=wjVg/PLOSOne_formatting_sample_main_body.pdf and https://journals.plos.org/plosone/s/file?id=ba62/PLOSOne_formatting_sample_title_authors_affiliations.pdf

Reply: We have checked the format and made adjustments if necessary. 

2. Please review your reference list to ensure that it is complete and correct. If you have cited papers that have been retracted, please include the rationale for doing so in the manuscript text, or remove these references and replace them with relevant current references. Any changes to the reference list should be mentioned in the rebuttal letter that accompanies your revised manuscript. If you need to cite a retracted article, indicate the article’s retracted status in the References list and also include a citation and full reference for the retraction notice.

Reply: We have checked the entire reference list and made adjustments if necessary. 

3. Please provide additional details regarding participant consent. In the ethics statement in the Methods and online submission information, please ensure that you have specified (i) whether consent was informed and (ii) what type you obtained (for instance, written or verbal, and if verbal, how it was documented and witnessed). If your study included minors, state whether you obtained consent from parents or guardians. If the need for consent was waived by the ethics committee, please include this information.

Reply: We have added information to the methods section to describe the informed consent procedure as clearly as possible. 

Revised sentences:

Ethics statement

This study was approved by the Medical Ethical Committee of the University of Maastricht (METC 2018-0698 and 2019-1035). Invitees were previous participants in the ‘Dutch Health Monitor’, which is a population-based survey of the Public Health Services South and North Limburg. Invitations were only sent to participants who had given permission (and their email-address) in the Dutch health monitor to be invited for future research. Before starting the questionnaire of the current SaNAE study, participants first gave electronic informed consent.

a. If you are reporting a retrospective study of medical records or archived samples, please ensure that you have discussed whether all data were fully anonymized before you accessed them and/or whether the IRB or ethics committee waived the requirement for informed consent. If patients provided informed written consent to have data from their medical records used in research, please include this information. 

Reply: This is not applicable

4. We note that you have included the phrase “data not shown” in your manuscript. Unfortunately, this does not meet our data sharing requirements. PLOS does not permit references to inaccessible data. We require that authors provide all relevant data within the paper, Supporting Information files, or in an acceptable, public repository. Please add a citation to support this phrase or upload the data that corresponds with these findings to a stable repository (such as Figshare or Dryad) and provide and URLs, DOIs, or accession numbers that may be used to access these data. Or, if the data are not a core part of the research being presented in your study, we ask that you remove the phrase that refers to these data.

Reply: Indeed, the results are already in the paper. We rephrased the sentence and referred to table 3. Revised sentence: While the first possibility (fear) was not measured in our study, the latter two were considered unlikely to explain our results, as both multivariate models were adjusted for respondents’ self-reported respiratory infections.

5. We note that you have indicated that data from this study are available upon request. PLOS only allows data to be available upon request if there are legal or ethical restrictions on sharing data publicly. For information on unacceptable data access restrictions, please see http://journals.plos.org/plosone/s/data-availability#loc-unacceptable-data-access-restrictions.

In your revised cover letter, please address the following prompts:

b. If there are ethical or legal restrictions on sharing a de-identified data set, please explain them in detail (e.g., data contain potentially identifying or sensitive patient information) and who has imposed them (e.g., an ethics committee). Please also provide contact information for a data access committee, ethics committee, or other institutional body to which data requests may be sent.

Data Availability Statement: Data in this study contain potentially identifying and sensitive patient information. Due to the General Data Protection Regulation, it is not allowed to distribute or share any personal data that can be traced back (direct or indirect) to an individual. Moreover, publicly sharing the data would not be in accordance with participant consent for this study. Therefore, interested researchers should contact the head of the data-archiving of the Public Health Service South Limburg (Helen Sijstermans: helen.sijstermans@ggdzl.nl) when they would like to re-use data.

c. If there are no restrictions, please upload the minimal anonymized data set necessary to replicate your study findings as either Supporting Information files or to a stable, public repository and provide us with the relevant URLs, DOIs, or accession numbers. Please see http://www.bmj.com/content/340/bmj.c181.long for guidelines on how to de-identify and prepare clinical data for publication. For a list of acceptable repositories, please see http://journals.plos.org/plosone/s/data-availability#loc-recommended-repositories.

We will update your Data Availability statement on your behalf to reflect the information you provide.

[Note: HTML markup is below. Please do not edit.]

Reviewers' comments:

Reviewer's Responses to Questions

Comments to the Author

________________________________________

Review Comments to the Author

Reviewer #1: In this study, Steijvers et al. explored the role of social networks in non-pharmaceutical infection preventive behaviours, especially for respiratory infectious diseases. By collecting data from a very large sample in the pre-COVID era, authors reported that to practice preventive behaviours was independently increased by social network aspects that reflected ‘weak relationships’. The study performed a through analysis of different social network aspects and I believe that the analysis is properly performed. Only few points need to be addressed before the paper can be suitable for publication.

1. Introduction is long. It can be summarizes by deleting the “The composition of social networks can be […] among middle aged and older adults in the Netherlands” paragraph;

Reply: We agree that the introduction is long and have shortened several paragraphs. However, we choose to not completely delete the section on network composition and the theory of strong and weak ties, because this is an important part of the introduction. This study is one of the first that aimed to investigate multiple social network characteristics (both structural (size, type of relation, proximity, mode of contact) and functional aspects (support)) and defined strong and weak relationships based on these characteristics. It is therefore of utmost importance that first a proper description of the social network characteristics is given. In addition, the theory of strong and weak ties is explained. This theory explains that especially the weak ties (geographically, practically, or emotionally more distant relationships) are important in providing informational support. Giving the reader a detailed description of the characteristics and theory in the introduction, contributes to a better understanding of defining the network characteristics into strong or weak relationships, and better interpretation of the found associations.

2. Statistical analysis methods are not clear. I would kindly ask to rephrase the whole paragraph as to better define, for each model the dependent and independent variable for sake of clarity. Also, it is not clear to me why authors entered in a step-wise regression some variables resulting from the previous model, and other ‘confounding founders’. What are these factors? How you chose them?

Reply: We agree that the statistical analyses paragraph can be written more clearly. See below for the revised text.

Revised paragraph:

Participants with missing values on any of the outcome or other variables (n=16) were excluded in analyses. Ordinal logistic regression analyses were performed for the count variable of preventive behaviors as outcome and the network characteristics as independent variables. Binary logistic regression analyses were performed for the four preventive behaviors (four outcomes) separately, with the network characteristics as independent variables, to gain more detailed insight into the specific behaviors. Multivariate logistic (for the separate preventive behaviors) and multivariate ordinal (for the count variable on behaviors) regression analyses were performed, calculating odds ratios (OR) with 95% confidence intervals. Multivariate model (I) included as independent variables the social network characteristics, and confounders (see below). Then, a multivariate model (II) was created including all social network aspects that were associated with the outcome (by a p-value <0.05) in model I and including confounders. To obtain the final model (II), we used a stepwise backward method for the social network variables (while keeping the confounders in the model) [25]. Confounding variables were sociodemographic factors (sex, age, educational level) [26–29], chronic conditions (depression, type II diabetes mellitus, cardiovascular diseases, asthma/COPD) [24,30–35], lifestyle factors (BMI, current smoking) [5,36] and self-reported upper and lower respiratory infections in the previous two months [25,37], as these variables previously were found to be associated with the dependent and independent variables. A priori, collinearity between network aspects was checked and ruled out (all correlations <0.66). A p-value < 0.05 was considered statistically significant. All analyses were performed using IBM SPSS Statistics (version 26.0).

3. In table 2, I do not foresee any advantage in reporting different levels of p-value. As author stated in the statistical section that 0.05 is the statistical significance value they should only report significant vs non-significant values;

Reply: We agree that the confidence intervals around the risk estimates are most informative, and thus we presented these in the Tables. We also included the p-values in the tables to provide the supporting statistics for additional clarification, and therefore like to keep these in the Tables. We agree that the description in the manuscript text in the results section could be more concise and there we reported only significant vs non-significant values in the results section. 

Reviewer #2: Interesting and innovative article showing how social networks potentially can increase the effectiveness and can promote healthy behaviors, taking advantage from social relationship and experience from peers. Social media can help people with health concerns or chronic disease, finding in Web communities a comforting source for information exchange, debate, and knowledge enrichment. I suggest the authors to implement Discussion with previous reports demonstrating this value (see Lavorgna L et al. Interact J Med Res. 2017) for health care organizations and beneficial repercussion reaching institutional stakeholders.

Resort to on-line survey has been widely amplified during pandemic due to need of reach a large population with a digital approach. Authors could emphasize the importance and usefulness of this easy and well accepted tool in the Conclusion.

See below for revised text: 

Specifically, the strengthening of the so called ‘weak relationships’, as the modes of interactions that provide informational support, that do not have to be in-person, thus possibly via online channels, is worth to be explored further. Such online contact opportunities may especially be useful in the current situation, where the COVID-19 pandemic led to widespread application of isolation, quarantine, and physical distancing, already likely shifting the use of these modes of interactions. Previous studies have shown that the internet can be used to receive social support from or social interaction with others [30,31].This reasoning leads to exploring the promotion of new/linking to already existing online, interactive, informal information platforms that allow people to exchange information and advice. Existing public health websites may be included because these provide information about the reasons why preventive behavior can be important and how to apply preventive measures; interacting with social network members contributes to increasing awareness of one’s behavior. Health care organizations could play an important role in such platforms, as they can facilitate validated information [31]. Future studies need to investigate how to promote such interactive, online platforms.

Attachment Submitted filename: Response to Reviewers.docx Click here for additional data file.

# Editor report: Decision Letter 1

**DOI:** 10.1371/journal.pone.0251862.r003

**Authors:** Luigi Lavorgna

5 May 2021

Social networks and infectious diseases prevention behavior: a cross-sectional study in people aged 40 years and older

PONE-D-21-06252R1

We’re pleased to inform you that your manuscript has been judged scientifically suitable for publication and will be formally accepted for publication once it meets all outstanding technical requirements.

Within one week, you’ll receive an e-mail detailing the required amendments. When these have been addressed, you’ll receive a formal acceptance letter and your manuscript will be scheduled for publication.

An invoice for payment will follow shortly after the formal acceptance. To ensure an efficient process, please log into Editorial Manager at http://www.editorialmanager.com/pone/, click the 'Update My Information' link at the top of the page, and double check that your user information is up-to-date. If you have any billing related questions, please contact our Author Billing department directly at authorbilling@plos.org.

If your institution or institutions have a press office, please notify them about your upcoming paper to help maximize its impact. If they’ll be preparing press materials, please inform our press team as soon as possible -- no later than 48 hours after receiving the formal acceptance. Your manuscript will remain under strict press embargo until 2 pm Eastern Time on the date of publication. For more information, please contact onepress@plos.org.

Kind regards,

Luigi Lavorgna

Academic Editor

PLOS ONE

# Editor report: Acceptance letter

**DOI:** 10.1371/journal.pone.0251862.r004

**Authors:** Luigi Lavorgna

10 May 2021

PONE-D-21-06252R1 

Social networks and infectious diseases prevention behavior: a cross-sectional study in people aged 40 years and older 

Dear Dr. Steijvers:

I'm pleased to inform you that your manuscript has been deemed suitable for publication in PLOS ONE. Congratulations! Your manuscript is now with our production department. 

If your institution or institutions have a press office, please let them know about your upcoming paper now to help maximize its impact. If they'll be preparing press materials, please inform our press team within the next 48 hours. Your manuscript will remain under strict press embargo until 2 pm Eastern Time on the date of publication. For more information please contact onepress@plos.org.

If we can help with anything else, please email us at plosone@plos.org. 

Thank you for submitting your work to PLOS ONE and supporting open access. 

Kind regards, 

PLOS ONE Editorial Office Staff

on behalf of

Dr. Luigi Lavorgna 

Academic Editor

PLOS ONE
